# The impact of physicians' communication styles on evaluation of physicians and information processing: A randomized study with simulated video consultations on contraception with an intrauterine device

**DOI:** 10.1111/hex.12521

**Published:** 2016-11-18

**Authors:** Martina Bientzle, Tim Fissler, Ulrike Cress, Joachim Kimmerle

**Affiliations:** ^1^ Leibniz‐Institut fuer Wissensmedien Tuebingen Germany; ^2^ Department of Psychology University of Tuebingen Tuebingen Germany

**Keywords:** attitudes, communication, decision making, information processing, intrauterine device, knowledge, physician attributes

## Abstract

**Objective:**

This study aimed at examining the impact of different types of physicians' communication styles on people's subsequent evaluation of physician attributes as well as on their information processing, attitude and decision making.

**Method:**

In a between‐group experiment, 80 participants watched one of three videos in which a gynaecologist displayed a particular communication style in a consultation situation on contraception with an intrauterine device. We compared doctor‐centred communication (DCC) vs patient‐centred communication (PCC) vs patient‐centred communication with need‐orientation (PCC‐N).

**Results:**

In the PCC condition, participants perceived the physician to be more empathetic and more competent than in the DCC condition. In the DCC condition, participants showed less attitude change compared to the other conditions. In the PCC‐N condition, the physician was perceived as more empathetic and more socially competent than in the other conditions. However, participants acquired less knowledge in the PCC‐N condition.

**Conclusion:**

We conclude that appropriate application of particular communication styles depends on specific consultation goals. Our results suggest that patients' needs should be addressed if the main goal is to build a good relationship, whereas a traditional PCC style appears to be more effective in communicating factual information.

## 1 INTRODUCTION

Major goals of physician‐patient communication are the establishment of a positive relationship between doctor and patient, and pertinent knowledge exchange.^1^, ^2^ A positive relationship between doctors and patients and a patient's positive impression of the physician have been shown to enhance patients' willingness to participate in therapy and stay the course.^3^ This also applies to the issue of contraception and family planning.^4^ All patients and seekers of medical advice should be informed about contraception methods in a pleasant communication atmosphere and should have the opportunity to make an informed choice regarding their preferred contraceptive. One approach that is not yet widely known, despite strong evidence that this is a very reliable method,^5^, ^6^, ^7^ is contraception with a frameless intrauterine device (IUD) with copper. Accordingly, consultations on contraception with a copper IUD are in the focus of the research presented here.

In addition, physicians' communication styles have an impact on patients' satisfaction^2^, ^8^ and even on physical outcomes such as patients' blood pressure or pain.^9^ Little is known, however, about how a physician's ability to explicitly take individual patient's needs into account affects patients' perceptions of their consultations and their acquisition of knowledge.

In the following sections, we provide an overview of research into physician‐patient communication before focusing on three specific types of physicians' communication styles: (i) doctor‐centred communication (DCC), (ii) patient‐centred communication (PCC) and (iii) patient‐centred communication with need‐orientation (PCC‐N). We then present our hypotheses regarding how these three styles will affect patients' perceptions of their consultations and their acquisition of knowledge.

### 1.1 Doctor‐patient communication

Effective communication between medical doctors and patients is a crucial part of successful medical care. Mazor et al.^10^ found that nearly half of the patients who report difficulties in their medical treatment complain about communication issues. Patients have several basic expectations in the communication with their physician. They expect doctors to (i) ask patients about their concerns and take their input seriously; (ii) try to understand the patient's overall situation and how their life is affected; (iii) explain things in everyday speech; (iv) aim at building a co‐operative relationship in terms of coming to an agreement about goals of treatment and priorities; and (v) strive towards a good relationship in that they are respectful and responsive and show sympathy.^11^, ^12^ Accordingly, many authors emphasize that physicians should take these aspects into account and support their patients in dealing with insecurities and address their feelings.^13^, ^14^, ^15^

### 1.2 Doctor‐centred and patient‐centered communication

Doctor‐centred communication is a conversation style that is based on the biomedical model of evidence‐based medicine.^15^ This communication style is characterized by a rational‐cognitive proceeding that does not pay much attention to a patient's individual feelings or concerns. In *PCC*, in contrast, physicians try to take the patient's individual values and demands into consideration. However, patient‐centredness is not described in a clear and consistent way in the literature. It is discussed as a quite fuzzy^16^ and complex concept. Recently, Scholl et al.^17^ have developed an integrative model of patient‐centredness that proposes 15 distinct but inter‐related dimensions, such as the communication between doctor and patient, a biopsychosocial perspective, patient involvement and emotional support. PCC can be characterized by an open, non‐directive conversation where the patients play an active role and where the physician takes their psychological situation and their social environment into account.^18^ Key features of PCC are openness towards the patients, usage of intelligible language, inclusion of patients and consideration of patients' feelings.^19^, ^20^

So, whereas in DCC physicians tend to ask closed questions and focus on the disease and on physical symptoms, in PCC they ask open questions and examine both the disease and how it is experienced by the patients.^21^ Physicians in PCC aim at actively including the patient in the conversation and taking psycho‐social aspects into account.^18^ Moreover, those physicians try to minimize medical jargon and check patients' understanding. Physicians in PCC consider their patients' expectations, integrate patients into the planning of their medical treatment and avoid interrupting them.^21^ In addition, patient‐centred physicians listen to patients' objections and concerns, encourage patients to participate in decision making and aim to achieve mutual agreement.^13^ Previous studies have found that PCC has various positive effects on patients, such as greater trust.^22^ In particular, PCC has a positive impact on patient satisfaction^23^. A recent study also found that physicians with high PCC scores were rated as more competent and more trustworthy.^8^ What previous research has hardly considered, however, is the impact of physicians' communication style on patients' acquisition of relevant medical knowledge in a consultation.

### 1.3 Patient‐centred communication with need‐orientation

A PCC‐N style largely features the same characteristics as PCC, but, in addition, pays extra attention to patients' particular needs and therefore could be even more suitable for meeting patients' expectations than “traditional” PCC.^2^, ^11^, ^12^, ^24^ Physicians with a PCC‐N style aim at recognizing and naming the particular needs of their patients in a given situation and at being particularly empathetic. Expressing and specifying a patient's needs allow for mutual understanding between doctor and patient of what is relevant and important for this patient in a medical consultation.

In PCC‐N physicians require the skill of detecting the needs, attitudes, values and intentions of their patients in a consultation situation. They empathize with those needs and articulate them.^25^ Recognizing patients' needs is important for physicians because this allows them to adapt their communication to individual demands.^26^ Recognizing those needs is also important in enabling physicians to take the needs of a patient into consideration when recommending an intervention. When health‐related information meets patients' needs, they tend to attribute more importance to this information.^27^ In addition, patients are influenced in their attitudes towards the particular medical procedures^28^ and are more willing to participate in prevention activities^29^ when medical information meets their needs. The individual needs that patients bring with them also have an impact on how they process medical information.^27^ Accordingly, PCC‐N and empathetic communication is occasionally already applied in various everyday health‐care situations and in medical education.^25^, ^30^

However, in previous empirical studies, PCC and PCC‐N have hardly been examined separately, and so it is largely unclear whether particular effects are the consequence of a PCC style (in contrast to DCC), or if they result indeed from explicitly addressing patients' and advice seekers' needs. In the study presented here, we aimed to examine the impact of physicians' communication style (DCC vs PCC vs PCC‐N) on various aspects of a patient's resulting evaluation of physicians, information processing and decision making. In particular, the study aimed at analysing the impact of the communication style in a medical consultation about contraception with a frameless copper IUD on participants' evaluation of a physician's *empathy*,* social competence* and *professional competence*, as well as the impact on participants' *knowledge acquisition*,* attitude change* and *decision modification*.

### 1.4 Hypotheses

Against the background of the considerations outlined so far, we assumed that a physician with a PCC style would be perceived to be more empathetic and more (socially and professionally) competent than a physician with a DCC style. We also assumed that PCC compared to DCC would result in the patient's acquiring more knowledge about the subject of the consultation. As patients tend to attribute more relevance to information when it is customized to their personal requirements,^31^, ^32^ we assumed that compared to DCC, PCC would result in a more positive attitude towards contraception with a frameless copper IUD when the physician also showed a positive attitude towards this method. Finally, we assumed that with PCC people would be more willing to make a decision in favour of a frameless copper IUD than with DCC. In addition, we took the overall positive picture of need‐orientation in medical consultations into account and, accordingly, assumed that PCC‐N would have even stronger effects on all of these outcome variables than PCC.

In sum, we stated the following hypotheses:

H1: Empathy: PCC‐N>PCC>DCC
H2: Social competence: PCC‐N>PCC>DCC
H3: Professional competence: PCC‐N>PCC>DCC
H4: Knowledge acquisition: PCC‐N>PCC>DCC
H5: Attitude change: PCC‐N>PCC>DCC
H6: Decision modification: PCC‐N>PCC>DCC

## 2 METHODS

### 2.1 Study design and participants

This study was designed as a three‐arm randomized controlled trial comparing the impact of DCC, PCC and PCC‐N communication styles.

The participants were recruited via e‐mail from the participant database of the Leibniz‐Institut fuer Wissensmedien in Tuebingen, Germany. To recruit participants, we used the Online Recruitment System for Economic Experiments (ORSEE).^33^ Eligibility criteria were the following: female, aged 18‐30 years, very good knowledge of the German language. All participants who registered for the study met these inclusion criteria. Because the study was conducted in the laboratory, all participants lived in Tuebingen or its surroundings.

The intended sample size was determined by power analyses for ANOVAs with α=.05, an intended power of 90% and a large effect size of *f*=0.42. This ex ante power analysis was undertaken to ensure a sufficiently large sample of participants for testing the hypotheses. Accordingly, 80 female participants were recruited for this experiment. Participation took about 1 hour and was compensated with eight Euros. Twenty‐seven participants were randomly assigned to the DCC, 27 to the PCC and 26 to the PCC‐N conditions through a computer‐generated randomization procedure. The investigators did not know which participants were allocated to the particular conditions, and the participants were not aware of the existence of the other conditions.

### 2.2 Procedure

To eliminate the influence of anxiety on the evaluation of communication style,^18^ we decided to use contraception as a subject matter as this topic is neither particularly threatening nor distressing. In addition, the importance of informed decision making is particularly emphasized in this area,^34^ so that providing balanced information is key. In a first step, we conducted a pre‐study with 30 female participants (age range: 19‐24 years, *M*=21.97, SD=1.77) to test our scales and to identify needs that are particularly important in such a consultation situation.

Initially, the participants in the main study were informed that they would participate in a study on the perception of information about contraception with a frameless copper IUD. All participants gave written informed consent. Then they filled in three pre‐test questionnaires, one regarding their *knowledge* about contraception with a frameless copper IUD, one regarding their *attitude* towards this device and one regarding their *decision* whether to use a frameless copper IUD in the near future. Subsequently, they were instructed to put themselves in the patient's place in a consultation about contraception with a frameless copper IUD. The participants were told that this patient had heard about the frameless copper IUD from a friend and wanted to be informed about this method by her gynaecologist. The patient had no concrete plans so far whether or not she wanted to use this contraception method herself. The participants were then asked to rate the importance of four different needs that were found in the pre‐study (see below) to be relevant in such a consultation. Next they watched a video presenting such a consultation with a gynaecologist. In a between‐group design, the participants were randomly assigned to one of three experimental conditions: they watched one of the three videos that showed a consultation with a gynaecologist who used either a DCC, PCC or PCC‐N style (see material section for details).

After watching the videos, participants filled in a post‐test questionnaire that contained manipulation check measures and also asked for the perceived *empathy* of the gynaecologist as well as her *social competence* and *professional competence*. In addition, this questionnaire asked once again for participants' *knowledge*,* attitude* and *decision*. Finally, we captured demographic data and debriefed the participants.

### 2.3 Material

To test the particular effects of different communication styles on the outcome variables, we decided to use video vignettes. This is an approved and valid method to investigate communication in medical consultations.^26^, ^35^, ^36^, ^37^ We scripted and filmed three videos. All of the videos showed a female patient in her mid‐twenties (played in every video by the same actress) who was seeking advice from a female gynaecologist (in her forties; played in all of the videos by the same actress) regarding contraception with a frameless copper IUD. In every video, the conversation was filmed as an over‐the‐shoulder shot from the patient's perspective to help the participants imagine themselves in her place.^38^ One video showed a consultation with a gynaecologist who used a DCC style; another video a gynaecologist with a PCC style; and a third video a gynaecologist with a PCC‐N style. All of the videos included the identical factual, evidence‐based information on the frameless copper IUD contraception method.^5^, ^6^, ^7^, ^39^

In the PCC and in the PCC‐N video (in contrast to the DCC condition), the gynaecologist did not interrupt the patient, avoided medical jargon, took the patient's private circumstances into consideration, asked open questions and asked the patient for concerns. In the PCC‐N condition, in addition, the physician asked actively after the patient's feelings and needs and aimed at recognizing and explicitly naming those needs.

All of the videos consisted of six scenes including the reception of the patient (scene 1) and the goodbye (scene 6), both of which were identical in every video. In each of the other four scenes, the patient expressed one specific need each. These four needs had been identified in the pre‐study as being particularly important in such a consultation situation: (i) clarity, (ii) the reliability of the contraception method, (iii) the patient's well‐being and (iv) the option for a future pregnancy. When those issues came up (eg reliability), the gynaecologist in the DCC condition reacted by only providing scientific information (eg “… the pearl index of the frameless copper IUD is even lower than that of the contraceptive pill”). In the PCC condition, she directly addressed the patient's feelings and then provided the same scientific information without medical jargon (eg “… you don't have be concerned … the frameless copper IUD is even safer than the contraceptive pill”). In the PCC‐N condition, the gynaecologist first explicitly named the patient's need, waited for the patient to confirm this and only then provided the same scientific information (eg “If I understand you correctly … it is very important for you that you can be sure about the reliability of the contraception method. [Waits for the patient to confirm.] So if it is about reliability … the frameless copper IUD is even safer than the contraceptive pill”). As a consequence of this explicit need‐orientation, the PCC‐N video was the longest in duration, followed by the PCC and the DCC video. Nevertheless, all of the videos included the identical factual information.

### 2.4 Measures

We measured how important the participants considered the four *needs* mentioned above (clarity, reliability, well‐being and future pregnancy) with three items each on seven‐point Likert scales. A sample item (capturing the need for reliability) was: “For me it is important that I can rely on the contraception method.”

We developed two scales as manipulation check measures: the first scale was designed to differentiate between the DCC condition and the two PCC conditions (*DCC scale*). With four items, we asked participants about typical DCC behaviour, such as the usage of technical terms by the physician or the physician's lack of interest in the patient as a person. These items were rated on four‐point Likert scales. The second scale was intended to differentiate between PCC and PCC‐N (*PCC‐N scale*). Here we asked participants to what extent the physician responded to the feelings and needs of the patient and whether the physician asked the patient if she wanted to receive further information before providing it. These two items were also rated on four‐point Likert scales.

For all of the following variables, we converted the single items via their common mean value into a joint value for each participant. Because the differing measures captured the answers on different scales, we standardized the score on the interval [0,1] to arrive at the same range of values for all measures. We measured perceived *empathy* by providing eight statements that participants had to rate according to how strongly they agreed with them on seven‐point Likert scales (standardized score [0,1]: 0*=total disagreement*; 1=*total agreement*). The statements were taken from Kim et al.^40^ Again, as while watching the video, participants were asked to put themselves into the patient's place. A sample item was: “The physician respected my feelings.”

We measured perceived *social competence* with ten pairs of adjectives that participants had to judge on nine‐point semantic differential scales, following the measurement by Willson and McNamara.^41^ Sample adjective pairs were: “friendly/unfriendly” and “polite/impolite” (standardized score [0,1]: 0=*total agreement with the “competent” adjective*; 1=*total agreement with the “incompetent” adjective*).

We measured perceived *professional competence* with seven adjective pairs that participants judged on nine‐point semantic differential scales, also according to the measurement by Willson and McNamara.^41^ Sample adjective pairs were: “experienced/inexperienced” and “accurate/inaccurate” (standardized score [0,1]: 0=*total agreement with the “competent” adjective*; 1=*total agreement with the “incompetent” adjective*).

We measured participants' *knowledge acquisition* by capturing their knowledge about contraception with a frameless copper IUD before and after the experiment. The knowledge test consisted of 11 statements about contraception with a frameless copper IUD (four statements were right; seven were wrong) and participants had to indicate whether a statement was right or wrong. An example of a wrong statement was: “The frameless copper IUD has to be replaced every 2 years.” Participants received one point each for correctly identifying a statement as right or wrong. All statements featured information that was provided in the videos. To determine participants' knowledge gain, we calculated the difference between their scores in the pre‐ and the post‐tests (standardized score [0,1]: 0=*no knowledge acquisition*; 1=*maximum knowledge acquisition*).

We measured participants' *attitude* change by capturing their attitude towards contraception with a frameless copper IUD before and after the experiment and by calculating the difference between their scores in the pre‐ and the post‐tests. The attitude test consisted of four pairs of adjectives that had to be judged on nine‐point semantic differential scales following the attitude measurement by Marteau et al.^1^ Participants had to indicate whether they considered the frameless copper IUD as a contraception method to be “advantageous/disadvantageous,” “unimportant/important,” “a good thing/a bad thing” and “inconvenient/convenient” (standardized score [0,1]: 0=*no attitude change*; 1=*maximum attitude change in favour of* (+) *or against* (−) *the IUD*).

To examine to what extent participants had modified their *decision* about whether to use a frameless copper IUD themselves, we captured their decision before and after the experiment and calculated the difference between their scores in the pre‐ and the post‐tests. This decision test is comprised of three items. Participants had to rate them on seven‐point Likert scales. A sample item was: “I have decided that I will use the frameless copper IUD as a contraception method as soon as possible” (standardized score [0,1]: 0=*no decision modification*; 1=*maximum decision modification in favour of* (+) *or against* (−) *the usage of the IUD*).

### 2.5 Ethical considerations

This research was performed in accordance with the Declaration of Helsinki and had full approval by the ethics committee of the Leibniz‐Institut fuer Wissensmedien (approval number: LEK2014/065). All participants participated voluntarily and gave written informed consent.

## 3 RESULTS

### 3.1 Participants

Eighty female participants were recruited for this experiment (age range: 18‐29 years, *M*=21.86, SD=2.20; 67 university students; 13 graduates). Three participants had to be excluded from the analysis because one participant knew one of the actresses in the videos personally, one specified that she was already using the frameless copper IUD as a contraception method and one indicated that all four needs were completely unimportant for her. All the following results refer to analyses of the remaining 77 participants, with 25 in the DCC, 26 in the PCC and 26 participants in the PCC‐N conditions.

### 3.2 Measurements

All scales demonstrated acceptable or good internal consistencies: need for clarity: α=.70; reliability: α=.91; well‐being: α=.67; future pregnancy: α=.78; DCC scale: α=.73; PCC‐N scale: α=.77; perceived empathy: α=.79; social competence: α=.95; professional competence: α=.85; attitude (pre): α=.76; attitude (post): α=.82; decision (pre): α=.73; decision (post): α=.65.

As expected, and consistent with the pre‐test, the participants assigned high levels of importance to the four needs that were brought up by the patient in the videos. Need for clarity: *M*=0.95, SD=0.07; reliability: *M*=0.93, SD=0.11; well‐being: *M*=0.95, SD=0.06; future pregnancy: *M*=0.87, SD=0.18. There were no significant differences among the three experimental conditions regarding participants' needs, their prior attitude towards contraception with a frameless copper IUD and their knowledge about the IUD, nor their decision regarding its usage, all *P*'s >.075. We found, however, that participants differed in their age among the conditions, *F*_2,74_=4.05, *P*=.021, and thus included age as a covariate (see below).

We found that our experimental manipulations were successful: the DCC scale differed among the experimental conditions, *F*_2,74_=71.87, *P*<.001, η^2^=.66, with higher scores in the DCC than in the two PCC conditions, as indicated by a contrast analysis. Regarding the PCC‐N scale, we also found significant differences, *F*_2,74_=79.14, *P*<.001, η^2^=.68, with higher scores in the PCC‐N condition.

### 3.3 Hypothesis testing

To test the hypotheses, we compared the mean differences among the three experimental conditions regarding the outcome variables using ANCOVAs with age as a covariate. For those outcome variables that yielded significant main effects, we provide post hoc tests with Bonferroni corrections to consider pairwise differences between the conditions. The means and standard deviations are shown in Table 1.

**Table 1:** Means and standard deviations of the outcome variables in the three experimental conditions

	DCC condition *M* (SD)	PCC condition *M* (SD)	PCC‐N condition *M* (SD)
Empathy	0.42 (0.15)	0.51 (0.13)	0.69 (0.09)
Social competence	0.51 (0.22)	0.72 (0.15)	0.84 (0.08)
Professional competence	0.65 (0.14)	0.80 (0.12)	0.84 (0.10)
Knowledge acquisition	0.72 (0.14)	0.73 (0.20)	0.62 (0.19)
Attitude change	−0.03 (0.19)	0.07 (0.18)	0.12 (0.16)
Decision modification	0.06 (0.17)	0.05 (0.16)	0.15 (0.18)

DCC, doctor‐centred communication; PCC, patient‐centred communication; PCC‐N, patient‐centred communication with need‐orientation.

In H1, we had assumed that *empathy* would differ among the three conditions, which was supported by the data, *F*_2,74_=31.19, *P*<.001, η^2^=.46. The DCC condition differed from the PCC condition, *P*=.022, and the PCC condition differed from the PCC‐N condition, *P*<.001.

Regarding *social competence* the data largely supported H2, *F*_2,74_=26.26, *P*<.001, η^2^=.42. The DCC condition differed from the PCC condition, *P*<.001, and the PCC condition tended to differ from the PCC‐N condition, *P*=.052.

In H3, we had assumed that *professional competence* would differ among the three conditions. We found a main effect for communication style, *F*_2,74_=16.58, *P*<.001, η^2^=.31. In particular, we found that participants considered the gynaecologist with the DCC style to be less competent than in the other conditions, both *P*'s <.001, whereas there was no difference between the PCC and the PCC‐N conditions, *P*=1.000.

The assumption of H4 was that *knowledge acquisition* would differ among the three conditions. We found indeed a main effect, *F*_2,74_=3.88, *P*=.025, η^2^=.10. The post hoc tests indicated a difference between the PCC and the PCC‐N conditions that was, however, contrary to the expected direction, with lower scores in the PCC‐N condition, *P*=.031. There were no differences among the other conditions, both *P*'s >.126.

The assumption of H5 was that there would be a difference among the three conditions in a change of *attitude*. We found a significant main effect, *F*_2,74_=5.79, *P*=.005, η^2^=.14. Post hoc tests indicated a significant difference between the DCC and the PCC (*P*=.017) as well as the PCC‐N conditions (*P*=.011), whereas there was no difference between the PCC and the PCC‐N conditions, *P*=1.000.

In H6, we had assumed that there would be a difference among the three conditions in *decision* modification. Even though the descriptive data seem to suggest higher scores in the PCC‐N condition (see Table 1), the hypothesis was not supported by the data, *F*_2,74_=2.08, *P*=.132.

## 4 DISCUSSION AND CONCLUSION

### 4.1 Discussion

This study examined the impact of physicians' communications styles on how patients subsequently evaluated various attributes of the physicians and how they processed information. We found that DCC, PCC and PCC‐N styles elicited differential effects. The presented study contributed especially to an understanding of the particular effects of explicitly addressing patients' needs. To our knowledge, this is the first study that has systematically investigated this factor in an experimental design. On the whole, it is apparent that participants evaluated the DCC style rather negatively. They perceived the gynaecologist with the DCC style to be the least empathetic and the least socially and professionally competent. We also found the least attitude change towards the recommended contraception method in the DCC condition. A patient‐centred approach to transmitting medical information seemed to be more convincing for laypeople.

Regarding the acquisition of knowledge, a PCC‐N style was the least appropriate in this study. One reason for this finding could be that many cognitive resources of the participants were occupied with the highly empathetic (and potentially less familiar) communication style. Regarding the evaluation of the physician (empathy, social and professional competence), the PCC‐N condition was superior to both the PCC and the DCC conditions, thereby extending previous findings.

Overall, the results should be handled with caution and cannot be generalized to a wide population. We tested our hypothesis with university students and graduates. Previous research has shown patients with a high level of education prefer a PCC style.^8^ Thus, it is unclear whether participants with a lower education level would have reacted to the video vignettes in the same way. Moreover, we have applied several measures that have been newly developed for the present study. Although these scales have a high level of face validity and showed acceptable reliability, we cannot be entirely sure about their psychometric properties. A statistical weakness of the study is that a set of multiple hypotheses was tested on the study population. We counteracted this issue by applying Bonferroni corrections. But, of course, there is still a remaining risk that problems of multiple comparisons have occurred. Another limitation is that the participants were only passive video viewers of a consultation. Even though this is an established method in patient education research,^26^, ^35^, ^36^, ^37^ future studies should also examine people's evaluation and information processing in real consultations.

### 4.2 Conclusion

It follows from the results of this study that it seems appropriate to address patients' needs in a medical consultation if the main goal is to build a good relationship and to establish trust. If the main goal of the consultation is to provide factual information, it might be more effective to use a “traditional” PCC style. Therefore, to use the ideal communication style in a consultation setting, physicians need to be aware of the main goal of the consultation as well as of the needs and feelings of the patient. Nevertheless, as this study was based on video vignettes and is also the first study to investigate explicitly the impact of addressing patients' needs, it is important to note that—without further studies—the results should be transferred to other contexts only very cautiously.

### 4.3 Practice implications

In a medical consultation situation, it is not only important what factual information is communicated but also the manner in which this is performed.^2^, ^42^ We found that especially a need‐orientation in PCC can improve the patient's evaluation of the physician, and this response could be important in establishing a trustful relationship between patient and physician. To meet a patient's needs, it is important to read between the lines of what a patient is saying, and to learn how to address individual needs. It is also essential that physicians be aware of the main objective of a consultation. They need to adapt their communication style to the situation in accordance with their goals.

## CONFLICT OF INTEREST

None.
